# Flowers and weeds: cell-type specific pruning in the developing visual thalamus

**DOI:** 10.1186/1741-7007-12-3

**Published:** 2014-01-25

**Authors:** Isabel Benjumeda, Manuel Molano-Mazón, Luis M Martinez

**Affiliations:** 1 Instituto de Neurociencias de Alicante, Avenida Ramón y Cajal S/N, 03550 Sant Joan d’Alacant, Spain

## Abstract

In the first weeks of vertebrate postnatal life, neural networks in the visual thalamus undergo activity-dependent refinement thought to be important for the development of functional vision. This process involves pruning of synaptic connections between retinal ganglion cells and excitatory thalamic neurons that relay signals on to visual areas of the cortex. A recent report in *Neural Development* shows that this does not occur in inhibitory neurons, questioning our current understanding of the development of mature neural circuits.

See research article: http://www.neuraldevelopment.com/content/8/1/24

## Standard model of neural circuit development

**Figure 1:**
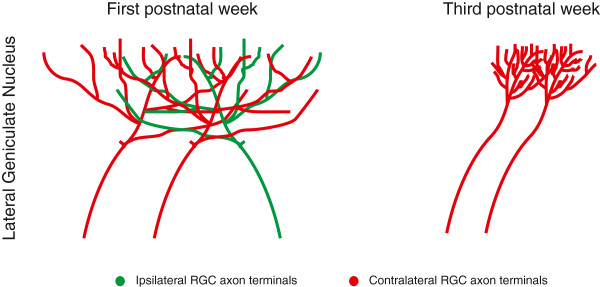
**Early postnatal pruning of retinal inputs to the thalamus.** During the first postnatal week (left), RGC axons find their appropriate target in the lateral geniculate nucleus following gradients of molecular guidance cues. Axons from both eyes are sparsely branched and cover large areas of the lateral geniculate nucleus where they overlap extensively. By the end of the third postnatal week (right), RGC axon terminals refine to increase the precision of the visual map, produce denser projections and segregate into eye-specific domains.

**Figure 2:**
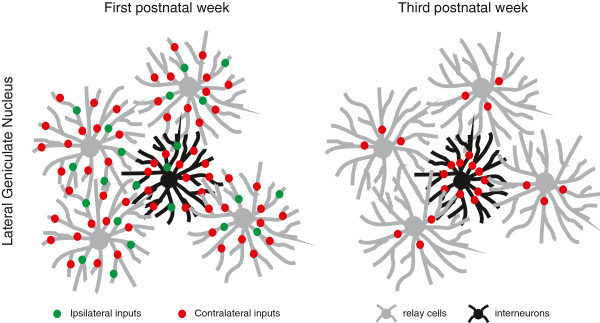
**Development of connectional specificity in the lateral geniculate nucleus.** In the first postnatal week (left), both relay cells (in gray) and interneurons (in black) receive a large number of retinal inputs originating in both eyes. By the end of the third postnatal week (right), and consistent with the refinement and eye segregation of RGC axons, the convergence onto relay cells is dramatically reduced and restricted to inputs coming from the same eye. At the same time, retinal synapses form in more proximal locations of the dendritic tree, increasing the efficiency of the afferent input. Inhibitory cells, on the other hand, maintain a large number of retinal inputs. If we consider that the cells depicted in the figure all sample from the same region of the retina, the difference in retinothalamic convergence between the relay cells and the interneurons would guaranty an equivalent coverage of visual space in the excitatory and inhibitory branches of the network.

The classical view on the formation and refinement of vertebral neural networks posits that activity-independent and -dependent mechanisms sequentially regulate different aspects of neural circuit development [1]. In line with this view, cell-type specification, axon pathfinding and targeting, and synaptogenesis have all consistently been shown to be under the control of genetically encoded programs, while axon terminal refinement (Figure 1) and connectional specificity (Figure 2) seem to be activity-dependent events [2]. In the visual system, a coarse but structured representation of the visual field, known as a retinotopic map, is established shortly after retinal axons reach their targets, the lateral geniculate nucleus of the thalamus (LGN) and the superior colliculus (SC), even in the absence of neural activity [2]. In contrast, other features of the LGN and SC local circuits, such as the spatial segregation of retinal projections into eye-specific domains, are still too diffusely organized at this initial developmental stage. At this point, before the onset of vision and up to a few days after eye opening, neighboring retinal ganglion cells (RGCs) in the immature retina generate spontaneous bursts of correlated action potentials [3]. These stereotyped waves of retinal spikes have recently been shown to coordinate patterned activity across multiple visual areas [4], where they are proposed to mediate the sculpting and refinement of retinotopic and eye-specific maps [3,5]. The local structure of retinal waves is ideal to provide an instructive role for precise retinotopy via synchronous signaling to strengthen synapses (a 'fire together, wire together’ mechanism known as Hebbian type reinforcement), while the asynchrony between the activities of both eyes could promote eye-specific segregation via activity-dependent competition [3].

## The role of activity dependent synaptic pruning

In keeping with this classic model, recent studies have shown that, during the first postnatal days, a single relay cell in the LGN can receive inputs from more than 20 RGCs located in either eye [5]. Over the period of retinal waves, most of these synapses are pruned away as RGC axon terminals recede and retinal convergence is dramatically reduced, reaching by the third postnatal week its mature state averaging three RGC inputs per LGN relay cell, all arising from the same eye [5] (Figure 2).

Also in support of the standard model of circuit development, when the normal patterns of endogenous retinal activity are altered, either using pharmacological manipulations or transgenic mice expressing the β2-n acetylcholine receptor in RGCs, the pruning of retinal axons is disrupted and the development of the cortical visual areas downstream is dramatically affected [3]. This has led to the proposal that retinal waves, with their particular spatiotemporal properties and binocular interactions, act as crude surrogates of natural visual stimuli to which cells in the visual system can respond by favoring some connections and abandoning others, even before the onset of visual experience. In this context, synaptic pruning would therefore be responsible for adapting the structure of local circuits to the properties of natural images through the elimination of metabolically costly neural connections that are not deemed functionally appropriate.

## Unknowns we know about

Notwithstanding its success in explaining the maturation and refinement of downstream circuits and sensory maps, our understanding of the underpinnings of this developmental model is still far from complete. First, we do not yet know the molecular pathways that mediate the axonal recession, spinogenesis and pruning subserving connectional specificity in the mature brain. Second, the activity-dependent learning rules that shape neural wiring, which include both Hebbian and non-Hebbian forms of plasticity, do not appear to be conserved across different brain regions. For instance, the dendritic trees of the principal cortical neurons increase in size, branching complexity and number of putative excitatory inputs, or spines, when we move along the processing hierarchy. This is particularly evident when we compare cells from the primary sensory areas, essential for detecting basic features in sensory stimuli, such as the orientation of a visual contour, to those in association regions and, finally, the prefrontal cortex, which are involved in higher level cognitive computations, like recognizing particular objects in a visual scene. These differences reflect a distinct change in the magnitude and possibly timing of spinogenesis and pruning in the different cortical areas [6]. Moreover, these different maturation profiles may even vary among different species. Third, computational models have recently shown that these activity-dependent learning rules might not even be required for certain features of receptive field, circuit and map development. Godfrey *et al*. [7] have found that axonal refinement in a model of the retinocollicular projection was independent of some of the spatiotemporal properties of retinal activity, including wave velocity, frequency and size. Furthermore, it has recently been proposed that the most salient organizing principles of the primary visual cortex, such as the presence of oriented receptive fields, columns and maps, could simply emerge by random, statistical sampling of inputs from a small patch of the RGC mosaics [8], without the need to invoke any activity-dependent wiring rule or interaction among cortical neurons. Random-wiring models instructed exclusively by gradients of molecular guidance cues have also been successful at simulating realistic swimming motor patterns, including the alternating left-right bursts of activity, in the spinal cord and hindbrain of *Xenopus* tadpoles [9]. These computational models suggest that molecular guidance cues and neural activity may, at least to some extent, play more overlapping and independent roles in neural development than previously thought.

## New finding

The reported large-scale reorganization of retinal synapses in the developing thalamus has always been studied from the perspective of the thalamocortical relay neurons. A recent report in *Neural Development*[10] now shows, for the first time, that in contrast to what is observed for the relay cells, the number of retinal inputs onto local inhibitory neurons remains constant in the LGN through the first five weeks of life. Thus, activity-dependent synaptic pruning, at least in the visual pathway, is not only area-specific but also cell-type specific. Given that the relative numbers and functional properties of excitatory and inhibitory neurons are largely conserved across different brain regions and even species, this result is likely to generalize to other sensory and motor systems of the brain, and raises two intriguing questions. First, what are the molecular mechanisms that mediate the effect of activity on synaptic remodeling in excitatory neurons that is absent in the inhibitory cells? A promising candidate suggested by the authors is the Ca^2+^ influx through L-type channels, which has been previously linked to the activation of CREB-related signaling cascades responsible for eye-specific segregation in the thalamus. Although inhibitory neurons also have L-channels, the plateau potential that results from their activation, and hence the influx of calcium, is more modest than in their excitatory neighbors. And, second, if synaptic pruning is required for adapting the LGN excitatory circuitry to the properties of the natural environment, why is it absent in the inhibitory branch of the network, which is widely regarded as fundamental for sharpening neuronal selectivity and improving perceptual discrimination in almost every other part of the brain?

## A complementary view on activity-dependent synaptic pruning

The resting membrane potential of interneurons is significantly closer to spike threshold than that of relay cells, which makes them respond more effectively to visual inputs. This might be necessary to keep in check the activity of excitatory neurons so they operate at regimes that are more energy efficient; but this advantage would come at the expense of limiting the percentage of inhibitory cells in the nucleus (20 to 25 %). As a consequence, they would require a larger retinal convergence to achieve an equivalent coverage of visual space as their excitatory counterparts (Figure 2). In principle, pruning of retinothalamic inputs onto relay cells would likely reduce the redundancy of the thalamic array, so that each cell responds to inputs from a distinct set of RGCs, giving a greater diversity of visual receptive fields overall. At the same time, having their inhibitory synaptic partners pool information from an overlapping but wider retinotopic area would increase the dynamic range of the thalamic relay, permitting the visual system to operate faithfully under very different light levels, such as in starlight and in bright sunlight. Both features are fundamental requirements for an optimal information code that, if proved to be true, could have far reaching consequences for our understanding of how the thalamus transforms the retinal message on its way to the cortex.

Therefore, if one thinks of the excitatory neurons as being of principal importance -the flowers of the nascent neuronal garden - the general scheme of the classical model of the early visual pathway development is likely to be correct. But results such as those of Seabrook *et al*. [10] show that inhibitory neurons - the weeds of the garden - might mature differently and their contribution is essential to achieve normal circuit function.

My garden is a balancing act between weeds and wonders.

Carol Stocker
